# Radiogenomics-based cancer prognosis in colorectal cancer

**DOI:** 10.1038/s41598-019-46286-6

**Published:** 2019-07-05

**Authors:** Bogdan Badic, Mathieu Hatt, Stephanie Durand, Catherine Le Jossic-Corcos, Brigitte Simon, Dimitris Visvikis, Laurent Corcos

**Affiliations:** 1 INSERM, UMR 1101, LaTIM, 22 rue Camille Desmoulins, 29238 Brest, France; 2 INSERM, UMR 1078, 22, avenue Camille Desmoulins, 29238 Brest, France

**Keywords:** Stem-cell research, Outcomes research

## Abstract

Radiogenomics aims at investigating the relationship between imaging radiomic features and gene expression alterations. This study addressed the potential prognostic complementary value of contrast enhanced computed tomography (CE-CT) radiomic features and gene expression data in primary colorectal cancers (CRC). Sixty-four patients underwent CT scans and radiomic features were extracted from the delineated tumor volume. Gene expression analysis of a small set of genes, previously identified as relevant for CRC, was conducted on surgical samples from the same tumors. The relationships between radiomic and gene expression data was assessed using the Kruskal–Wallis test. Multiple testing was not performed, as this was a pilot study. Cox regression was used to identify variables related to overall survival (OS) and progression free survival (PFS). *ABCC2* gene expression was correlated with N (p = 0.016) and M stages (p = 0.022). Expression changes of *ABCC2*, *CD166*, *CDKNV1* and *INHBB* genes exhibited significant correlations with some radiomic features. OS was associated with Ratio 3D Surface/volume (p = 0.022) and *ALDH1A1* expression (p = 0.042), whereas clinical stage (p = 0.004), *ABCC2* expression (p = 0.035), and Entropy_GLCM_E_ (p = 0.0031), were prognostic factors for PFS. Combining CE-CT radiomics with gene expression analysis and histopathological examination of primary CRC could provide higher prognostic stratification power, leading to improved patient management.

## Introduction

Colorectal carcinoma (CRC) is characterized by a marked intra-tumor genetic heterogeneity^1^, which may be, at least partly, assessed on medical images using radiomics analysis to quantitatively characterize a delineated tumor volume using various metrics^2^. Recent studies have investigated the prognostic value of computed tomography (CT)-derived radiomic features of patients with colorectal cancers^3–7^. In addition, high-throughput platforms have been developed with the aim of obtaining genomic and transcriptomic data to better characterize cancer at the molecular level^8^. Current clinical applications of genomic information use mutational data from *KRAS*, *NRAS* and *BRAF* genes, which are effectors of the *MAPK* (mitogen-activated protein kinase) pathway, as prognostic and predictive biomarkers for standard treatment, and the *HER2* gene for targeted therapies^9,10^.

In a broader sense, radiogenomics aims at investigating the relationship between imaging features and gene expression alterations, as well as their potential complementary value in predictive modeling^11^. To date, no studies have examined either the relationships between gene expression and radiomics image features of CRC, or their complementary prognostic value. To investigate this question, we selected a small set of genes previously identified as relevant for CRC and conducted a pilot study to explore their relationship and complementary prognostic value for overall (OS) and progression-free survival (PFS), in a retrospectively recruited cohort of 64 patients.

## Results

### Relationships between clinical, histopathological characteristics and gene expression profiles

The deregulation of gene expression observed in our cancer sample set showed good concordance with that observed in the TCGA samples (Table 1). Only *ABCC2* gene expression was correlated with UICC Stage (p = 0.028), N stage (p = 0.016) and M stage (p = 0.022). Age or gender were not correlated with gene expression profiles.

**Table 1:** Gene expression changes in colorectal carcinoma as compared to normal tissue in our cohort and similarly in 2 TCGA cohorts.

Gene Symbol	Our cohort		TCGA-Microarray			TCGA-RNAseq		
	Mean	SEM	Mean	SEM	p adj*	Mean	SEM	p adj*
*ABCB1*	0.451	0.05	0.332	0.021	<0.0001	0.447	0.026	<0.0001
*ABCC2*	4.211	1.03	3.45	0.333	<0.0005	16.625	1.69	<0.0001
*ABCG2*	0.113	0.01	0.074	0.008	<0.0001	0.05	0.005	<0.0001
*ALDH1A1*	0.667	0.08	0.579	0.043	<0.0001	0.574	0.044	<0.0001
*CD166 (ALCAM)*	1.722	0.12	1.89	0.086	<0.0001	1.955	0.057	<0.0001
*CDKN1A*	0.592	0.04	0.584	0.023	<0.0001	0.457	0.017	<0.0001
*INHBB*	1.599	0.23	4.217	0.533	<0.0001	6.88	0.645	<0.0001

^*^Comparison of CRC *versus* NT (Normal Tissues) was performed by Welch’s t-test and p values for TCGA data were corrected for multiple testing by Benjamini-Hochberg method (p.adjust function in R environment). SEM - Standard error of the mean.

### Relationships between radiomics and gene expression changes

Some of the first, second and third order radiomic features were significantly correlated with changes in gene expression, *e.g. ABCC2, CD166* (Kruskal-Wallis analysis). The expression changes in four genes (*ABCC2*, *CD166*, *CDKNV1*, *INHBB*) exhibited a significant correlation with radiomic features. Of note, the discretization method influenced the correlation with gene expression, *e.g. ABCC2* gene expression was correlated with Low gray level zone emphasis (LGLZE) and Small zone low gray emphasis (SZLGE) after **R** discretization but not after **L** or **E**.

### Overall relationships between clinical, histopathological, radiomics, gene expression profiles and outcome

Cox univariate analysis revealed histopathological (stage IV), *ALDH1A1* expression and several radiomic features (Ratio 3D Surface/volume, Flatness, Inverse difference moment (IDM_R_), Inverse difference (ID_R)_)) as predictive factors for overall survival (OS) (Table 2). Multivariate analysis identified Ratio 3D Surface/volume and *ALDH1A1* as independent prognostic factors. Their combination in Cox multivariate regression led to a HR of 8.4, compared to lower values of 2.8 and 3.3 alone, as well as for the stage alone (HR = 3.1). It should be emphasized however that significant overlap was observed between the 95% CI of these stratification results: [3.4–20.6] for the multivariate combination of Ratio and ALDH1A1, compared to [1–7.3], [1.2–9.2] and [1.1–8.6] for Ratio, ALDH1A1 and stage respectively.

**Table 2:** Overall survival and progression free survival Cox univariate analysis.

	Covariate	HR	95% CI	*p*-value
**Progression free survival**				
Histopathological	N = 1	7.8	1.7 to 35.0	0.0076
	Stage = 3	11.1	2.1 to 57.9	0.0044
Gene expression	*ABCC2* (unchanged)	5.0	1.1 to 22.4	0.0354
Radiomic	Flatness	0.0003	0.0 to 0.3	0.0234
	SENT_GLMC R_	0.5	0.31 to 0.91	0.0205
	Entropy_GLMC E_	0.046	0.006 to 0.35	0.0031
	GLNU_L_	0.074	0.007 to 0.77	0.0298
**Overall survival**				
Histopathological	Stage = 4	3.1	1.1 to 8.6	0.0304
Gene expression	*ALDH1A1* (up-regulated)	2.8	1.0 to 7.3	0.0420
Radiomic	Ratio 3D Surface/volume	3.3	1.2 to 9.2	0.0219
	Flatness	0.012	0.0001 to 0.6	0.0275
	IDM_R_	270.1	1.3 to 55079.2	0.0401
	ID_R_	655.3	1.1 to 393927.0	0.0481

HR = hazard ratio. SENT (sum entropy). Entropy_GLCM_ = entropy from grey-level-co-occurrence-matrix. GLNU = grey-level nonuniformity. IDM = Inverse difference moment. ID = Inverse difference.

**Figure 1:**
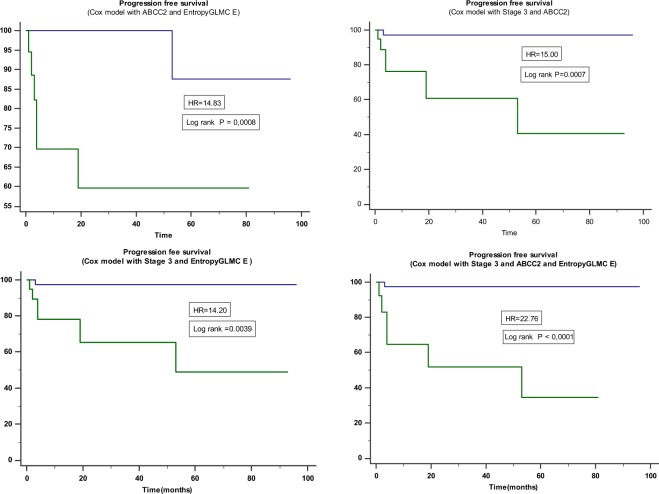
Kaplan-Meier analysis of PFS with Cox models combining Stage III, *ABCC2* expression and Entropy_GLMC E_ led to increased stratification power.

Some radiomic features, namely Flatness, Sum entropy (SENT_R_), entropy from Grey-level-co-occurrence-matrix (Entropy_GLCM E_), Grey-level non-uniformity (GLNU_L_), *ABCC2* mRNA level, Stage III and node status (N) were significantly associated with PFS on Cox univariate analysis. On multivariate analysis, stage III, *ABCC2* and Entropy_GLCM E_ remained independent prognostic factors of PFS (p = 0.0001). Their combination through Cox modeling led to an HR above 22, compared to lower values when considered alone or two by two (Table 3; Fig. 1). Again, overlaps between the 95% CI of each HR were observed nonetheless.

**Table 3:** Kaplan-Meier analysis and resulting hazard ratios for stratifying patients for OS and PFS using single features identified by the univariate Cox modeling or their combination.

	HR	95% CI	*p-*value
**Model Overall survival (OS)**			
Ratio 3D Surface/volume	2.8	1.0 to 7.3	0.0420
*ALDH1A1*	3.3	1.2 to 9.2	0.0219
Cox model with Ratio and *ALDH1A1*	8.4	3.4 to 20.6	0.0005
**Model Progression free survival (PFS)**			
Stage 3 only	11.1	2.1 to 57.9	0.0044
Cox model combining Stage 3 and Entropy_GLMC E_	14.2	2.4 to 85.3	0.0039
Cox model combining Stage 3 and *ABCC2*	15.0	2.9 to 77.4	0.0007
Cox model combining *ABCC2* and Entropy_GLMC E_	14.8	2.9 to 76.2	0.0008
Cox model combining Stage 3, *ABCC2* and Entropy_GLMC E_	22.8	3.7 to 141	<0.0001

## Discussion

We report here a promising potential added value of combining some radiomic features derived from contrast-enhanced CT images with gene expression features for predicting disease outcome of patients with primary CRC.

The impact of chemotherapy in CRC patients is limited by the inter-individual variability in drug response and the acquisition of resistance in which ATP-binding cassette (ABC) transporters play a crucial role by increasing the efflux of anticancer drugs outside of cancer cells^12,13^. Significantly, higher *ABCC2* mRNA levels were previously observed in adenomas with mild to moderate dysplasia, as well as in carcinoma, whereas *ABCG2* mRNA levels were decreased^14^, similarly to the data presented here. We also found correlations between *ABCC2* mRNA levels and PFS of CRC patients. The rise in *ABCC2* mRNA in CRC could reflect a functional defect. By contrast, the strong drop in *ABCG2* mRNA was not associated with any other parameter. However, it seems possible that some other member of the family of ABC transporters could have compensated for this reduction, leaving it relevant as a marker, but with low or unappreciable effect on the response to drugs. A similar situation occurred for the *ABCB1, CDKNV1 or CDKNV2* genes. It remains, however, that these gene expression changes should be worth investigating further to determine how they contribute to characterizing, or possibly, participate in the establishment or maintenance of the cancerous phenotype.

ALDH1A1 is a detoxifying enzyme that confers resistance to alkylating chemotherapeutic agents and protects against oxidative damage by catalyzing the irreversible oxidization of cellular aldehydes^15^. High *ALDH1* expression indicates a poor prognosis in CRC patients that correlates with the T stage, N stage and tumor differentiation^16^. Overall survival was associated in our study with *ALDH1A1* gene expression on Cox univariate, but not multivariate analysis, probably due to the limited size of our cohort.

A number of CT-derived radiomic features have been linked to gene expression profiles, for example in lung cancer^2^. CE-CT features analysis used for preoperative tumor staging^5^ correlated with lymph nodes metastasis in CRC^17^. CE-CT radiomics analysis could differentiate high-grade from low-grade CRC^18^. Features derived from intensity histograms, including entropy, standard deviation and skewness, were associated with *KRAS* mutation and tumor grade in CRC liver metastases^4^. Progression free survival, but not overall survival, was correlated with entropy from GLCM in our study. This difference can be explained by the fact that first order statistics features describe the distribution of individual voxel values without concern for spatial relationships^19^.

In our study, some second and third order texture features were significantly associated with PFS and OS. The repeatability of these metrics could be affected by the discretization choices, but the discretization value chosen for this study (64 bins) has been shown previously to provide a good compromise^20^. Geometric features such as flatness, minor axis length or sphericity are known already to be reliable shape descriptors that can be related to the high repeatability of segmentation^21^. Some shape-based features were significantly associated with OS and PFS in our study.

As shown here, combining an increasing number of features improved the stratification of patients resulting in a nomogram combining clinical stage, gene expression (*ALDH1A1*, *ABCC2*) and CE-CT radiomic features, suggesting that complementary prognostic value could be obtained not only from histopathological examination, but also using the pre-therapeutic CT and gene expression features. Our nomogram could thus be used to identify patients with poor prognosis who could then be offered alternative treatment options, such as targeted therapies or treatment intensification, using baseline CE-CT images that are acquired routinely for staging in clinical management.

Our study has several limitations. First, our cohort is retrospective and from a single center. It is also limited by the number of patients and events, due to the fact that tumor tissue samples were available only for a fraction of the patients, and that funds for the expensive transcriptomic analysis were limited. This limited number of patients and events led to high 95% confidence intervals around the estimated HR values which limit the reliability of their comparison. Second, repeatability of radiomics data is important to guarantee reliable results and several texture features have already shown their repeatability in other cohorts and types of cancers^22,23^. Since only routine CE-CT acquisitions were available for our cohort, we did not perform a repeatability evaluation of texture features and their prognostic value. Nevertheless, we performed texture analysis on the entire tumor volume, despite the time-consuming nature of whole tumor analysis. By contrast, the use of the largest cross-sectional area rather than the whole tumor to extract texture features could be a limitation in some tumors given the overall aim to quantify heterogeneity^3^. Finally, only radiomics extracted from portal enhanced CT scans were used for this study. Some recent observations showed that features extracted from non-enhanced and contrast-enhanced CT provide complementary prognostic information, thus suggesting that analysis of this two modalities could better characterize CRC tumors^24^.

Combining CE-CT radiomics, gene expression analysis and histopathological examination of primary CRC demonstrated an association with survival, suggesting that this strategy could provide complementary prognostic value, which might be beneficial for therapeutic decisions. Further analyses of genomic mutations or rearrangements could also strengthen such multi-parameter approaches, provided all of this patient information can be obtained readily and be cost-efficient.

## Patients and Methods

### Patients

Between January 2008 and May 2017, 653 colorectal resections were performed at our institution. For 64 of them (36 M, 28 F; mean age 71 (range 26–93), 27 in the right colon, 3 in the transverse colon, 20 in the left colon and 14 in the rectum) all the data and material required for our retrospective investigation were available: clinical and histopathological data, CT scans, outcome information (date of diagnosis, surgery, progression or last follow-up and death) and tissue samples (from our local registered tumor tissue collection). According to the UICC staging, there were 12 stage I, 25 stage II, 15 stage III and 12 stage IV tumors. Informed consent was obtained from each participant included in the study. Tumor localization was determined by colonoscopy and the tumor was visible on CE-CT images. We excluded patients with previous chemotherapy or radiotherapy or tumors not confirmed by anatomopathological examination. We also excluded patients with tumors located in the medium and lower rectum, because of lower CT scan resolution preventing appropriate delineation of rectal tumors, and because of different treatment modalities between colon and rectum tumors. Additional patient characteristics, such as potential comorbidity conditions at time of diagnosis (drug absorption, tobacco exposure or alcohol consumption, etc.), were not available. This study was conducted after approval by Brest University Hospital institutional ethics committee.

**Figure 2:**
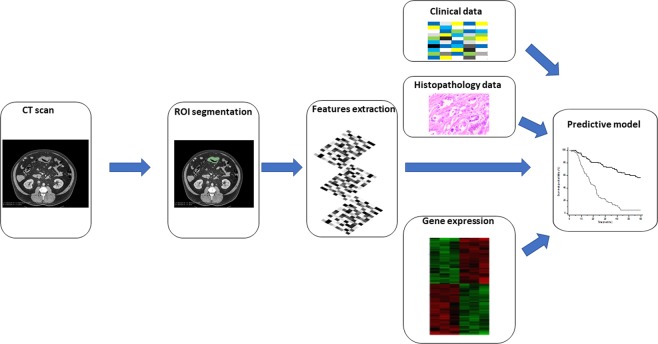
Workflow of prognosis model construction. A colorectal tumor is delineated in every slice and validated by an experienced physician. This allows creation of a 3D representation of the tumor. Radiomic features (intensity, shape, texture) are extracted from this delineated tumor, and integrated with clinical, histopathology and genomic/gene expression data.

A workflow of our study is displayed in Fig. 2.

### Imaging

Abdominal CE-CT assessment was performed as part of a routine imaging protocol at our institution before surgery or systemic treatment (radiotherapy/chemotherapy). Images were collected from the archive and communication system (PACS). The portal venous phase CT images were exploited for the present analysis because they allowed to clearly distinguish tumor tissue from adjacent normal bowel wall. Scans were performed on a Siemens Definition AS64 (Siemens medical, Erlangen, Germany). Standard acquisition settings were 120 kVp tube voltage, automatic tube current-exposure time product, 0.5 s rotation time, 1.25 mm slice thickness, matrix size of 512 × 512 pixels and inspiratory breath hold. Intravenous contrast (Xenetix 350, Guebert, Roissy, France) was administered at a 3 mL/sec injection rate with a pump injector (Medrad Stellant, Bayer, NY).

### Radiomics analysis

Primary tumors were semi-automatically delineated by one experienced physician using the 3D Slicer™ software^25^ on the portal phase CT images. Imaging Biomarkers Standardization Initiative (IBSI) compliant radiomic features were extracted from each segmented volume using an in-house software that was validated with the most up-to-date IBSI reference document and benchmark values^26^. For textural features, matrices were constructed in 3D according to the merging technique (Supplementary Material 1). No filter-based analysis (either textural features on wavelet decompositions^2^ or histogram analysis in sub-volumes identified by log of Laplacian filters^27^) was included to reduce the number of considered features and because these have not yet been included in the IBSI^24^.

The intensities in the original images were discretized for second and third order textural matrices’ calculations^28^. It has been shown that this step can have a significant impact on the texture values and distributions^28^. Three different sets of textural features were thus generated with different methods. The first two consisted of a discretization into 64 bins using either the fixed bin number approach (denoted from here onwards L) or histogram equalization, (denoted from here onwards E)^26,28^. The third one consisted in resampling the original grey-levels into a variable number of bins of fixed width (in our case 10 Hounsfield units (HU), denoted from here onwards R)^24,28^ (see Supplementary Material 1 for radiomics analysis).

### Tissue samples processing

Tissue samples were obtained from our institution tissue bank. H/E staining was performed for all samples, and the tumor content within cancer samples was above 80%. Repartition of low grade (stages 0, I and II) and high-grade carcinoma samples (stages III and IV) was homogeneous. The tissue fragments were stored in RNAlater® stabilization solution (Ambion, France), a reagent that prevents mRNA from degradation. Total RNA was extracted with the AllPrep DNA/RNA Mini kit (Qiagen, Courtaboeuf, France) from homogenized tissue samples (20 mg). RNA purity and integrity were evaluated by measuring the optical density ratio (A260/A280) and the RNA Integrity Number (RIN) using the RNA 6000 Nano LabChip and the 2100 BioAnalyzer (Agilent, Massy, France), respectively. Only RNA samples with a 28S/18S ratio >1.0 and RIN >5.0 were used. Reverse transcription and real-time PCR amplification were performed using conventional methods with reagents from Applied Biosystems (Applied Biosystems, France) and a StepOnePlus real time PCR system. The RNA levels were standardized to those of beta2-microglobulin, which was invariant between cancer and healthy tissue. The engineered PCR array plates were prepared at Eurogentec (Eurogentec, Belgium). We selected genes that have been previously reported for their expression changes in CRC (*ABCB1, ABCC2, ALDH1A1, CDKNV1, CDKNV2, INHBB, CD166 and ABCG2*) as compared to healthy tissue^14,29–31^. The gene characteristics are presented in Supplementary Table 1. To establish an independent validation set, we downloaded expression microarray and RNA-sequencing data from 221 and 358 CRC patients, respectively, from The Cancer Genome Atlas (TCGA) portal^32^ and the Broad GDAC Firehose^33^ by selecting the colorectal adenocarcinoma cohort (COADREAD). Patient characteristics from this cohort are presented in Supplementary Table 2.

### Statistical analysis

Statistical analyses were performed with MedCalc Software version 14.8.1. Comparisons between gene expression profiles and continuous variables were done using the non-parametric Kruskal-Wallis test. Comparisons between gene expression profiles and discrete variables were performed using the Chi2 test or Fisher’s exact test. Progression free survival (PFS) was defined as the time from diagnosis to first event (local or metastatic failure or death). Patients with no events were censored at the time of last follow-up. Overall survival (OS) was defined as the time from diagnosis to death from any disease-related cause or last follow-up. The Kaplan-Meier method and log-rank test with cut-off thresholds determined by receiver operating characteristic curve (ROC) analysis (according to Youden’s index) were used to determine the prognostic value of each feature. For each variable, relative risks were estimated using a univariate Cox model and expressed with their 95% confidence interval. Cox multivariate analysis was carried out using characteristics found significant in the univariate analysis. Statistical significance was set as p < 0.05 without correction for multiple testing as this is a hypothesis-generating study.

### Approval, accordance and informed consent

All procedures performed in studies involving human participants were in accordance with the ethical standards of the institutional and/or national research committee and with the 1964 Helsinki declaration and its later amendments or comparable ethical standards. Informed consent was obtained from all individual participants included in the study. All samples were included in a registered tumor tissue collection and the present study was conducted after approval by the CHU Brest institutional ethics committee^34^.

## Supplementary information

Supplementary material

## Data Availability

CT images used in this study can be made available on request for specific research purposes.
