# Supplementary material for: Radiogenomics-based cancer prognosis in colorectal cancer
Source: Sci Rep. 2019 Jul 5;9:9743. doi: 10.1038/s41598-019-46286-6 (PMC6611779; doi:10.1038/s41598-019-46286-6)
Supplement: Supplementary file 1 — Supplementary material [file 41598_2019_46286_MOESM1_ESM.docx]

**Radiogenomics-based cancer prognosis in colorectal cancer**

Bogdan Badic^1*^*,* Mathieu Hatt^1^, Stephanie Durand^2^, Catherine Le Jossic-Corcos^2^, Brigitte Simon^2^, Dimitris Visvikis^1§^, Laurent Corcos^2§^

**^1^**INSERM, UMR 1101, LaTIM, 22 rue Camille Desmoulins, 29238 Brest, France

**^2^**INSERM, UMR 1078, 22, avenue Camille Desmoulins 29238, Brest, France

## Supplementary material 1

## Radiomics analysis

For the purpose of this analysis, only primary tumors previously identified were analyzed. Primary tumors were semi-automatically delineated by one experienced physician using the 3D Slicer™ software (1) to manually delineate morphological tumor volumes in 3D on the portal phase CT images. Radiomics features were extracted in 3D from the segmented volumes. Features were implemented in-house following the most up-to-date guidelines from the Imaging Biomarkers Standardization Initiative (IBSI) (2). For textural features, matrices were constructed in 3D according to the merging technique. No filter-based analysis (either textural features on wavelet decompositions (3) or histogram-analysis in sub-volumes identified by log of Laplacian filters (4)) was included to reduce the number of considered features and because these have not yet been included in the IBSI(5).

The intensities in the native images were discretized for second and third order textural matrices’ calculations (6). It has been shown that this step can have a significant impact on the texture values and distributions (7). Three different sets of textural features were thus generated with different methods. The first two consisted of a discretization into 64 bins using either a uniform distribution of the original Hounsfield units (HU) (IBSI reference document, p. 12) or a histogram equalization (equation 1) (7). The third one consisted in resampling the original grey-levels into a variable number of bins of fixed width, in our case 10 HU (IBSI reference document, p.12)(5) (7).

$I_{E}(p)=1+\left( 64-1 \right)\times CH(p)$ (Equation 1)$CH is the cumulative histogram function$

These three different methods are denoted from here onwards as L, E and R.

**List of features and formulas** (8)

1. **Histogram-based (first order) metrics.**

The intensity histogram is generated by discretising the original set of grey levels X_gl_ into grey level bins. Let Xd = {Xd;1; Xd; _2_ ,…., Xd;Nv} be the set of Ng discretized grey levels of the Nv voxels in the ROI intensity mask. Let H = {n1; n2; ….; nNg} be the histogram with frequency count ni of each discretised grey level i in Xd. The occurrence probability pi for each grey level bin i is then approximated as *pi* = *ni*/*Nv*.

Mean is calculated as :

$$\sum_{i=1}^{Ng} i pi$$

Variance the spread or variation around the mean

$$\frac{1}{Nv}\sum_{k=1}^{Nv} \left( X_{gl,k}-\mu\right)^{2}$$

Skewness: symmetry of intensity values in an image. Skewness is zero if the histogram is symmetrical

$$\frac{\frac{1}{Nv}\sum_{k=1}^{Nv} \left( X_{gl,k}-\mu\right)^{3}}{\left( \frac{1}{Nv}\sum_{k=1}^{Nv} \left( X_{gl,k}-\mu\right)^{2} \right)^{\frac{3}{2}}}$$

Kurtosis: indication of histogram flatness

$$\frac{\sum_{i=1}^{Ng} \left( i-\mu\right)^{4}p_{i}}{\left( \sum_{i=1}^{Ng} \left( i-\mu\right)^{2}p_{i} \right)^{2}}-3$$

Energy: measures uniformity of intensity values

$$\sum_{k=1}^{N_{v}} X_{gl,k}^{2}$$

Entropy_HIST:_ The image entropy specifies the uncertainty in the image values. Measures the averaged amount of information required to encode the image values. (NOTE: We will differentiate between the various entropy calculations in this document, specifying the distribution from which the entropy is computed)

$$Ent=-\sum_{i=1}^{N_{g}} p_{i} \log_{2} p_{i}$$

1. **Grey-level co-occurrence matrix GLCM (also called grey tone spatial dependence matrix GTSDM).**

Let *p* be the normalized (sum all of matrix entries is one) Grey level co-occurrence matrix.

For the metrics calculations we use the following:

$$p_{x}\left( i \right)=\sum_{j=1}^{G_{max}} \left\{ p_{i,j} \right\} {; p}_{y}\left( j \right)=\sum_{j=1}^{G_{max}} \left\{ p_{i,j} \right\}$$

$$p_{x+y}\left( n \right)=\sum_{i+j=n} \left\{ p_{i,j} \right\} ; n\in\left\{ 2,3\ldots,2\cdot G_{max} \right\}$$

$$p_{x-y}\left( n \right)=\sum_{\left| i-j \right|=n} \left\{ p_{i,j} \right\} ; n\in\left\{ 0,1\ldots,G_{max}-1 \right\}$$

$$\mu_{x-y}=\sum_{n=0}^{G_{max}-1} \left\{ {n\cdot p}_{x-y}(n) \right\}$$

Angular Second Moment (ASM)

$$f_{1}=\sum_{i=1}^{N_{g}} \sum_{j=1}^{N_{g}} {p_{i,j}}^{2}$$

Contrast_GLCM_: measures the amount of local variation in intensity values

$$f_{2}=\sum_{i=1}^{N_{g}} \sum_{j=1}^{N_{g}} {(i-j)}^{2}p_{i,j}$$

Correlation: measures the linear dependencies of intensity values in an image

$$f_{3}=\frac{1}{\sigma_{i}\cdot\sigma_{j}}\sum_{i=1}^{N_{g}} \sum_{j=1}^{N_{g}} (i-\mu_{i})(j-\mu_{j})p_{i,j}$$

Inverse Different Moment (IDM). Inverse difference moment is similar in concept to the inverse difference feature, but with lower weights for elements that are further from the diagonal.

$$f_{4}=\sum_{i=1}^{Ng} \sum_{j=1}^{Ng} \frac{p_{ij}}{1+\left( i-j \right)^{2}}$$

Sum Average (SAVE). The sum average for the cross-diagonal probabilities

$$f_{5}=\sum_{k=2}^{2Ng} {kp}_{i+j,k}$$

Sum Variance (SVAR): The sum variance for the cross-diagonal probabilities

$$f_{6}=\sum_{k=2}^{2Ng} {\left( k-\mu\right)^{2}p}_{i+j,k}$$

GLCM Sum Entropy (SENT). The sum entropy for the cross-diagonal probabilities

$$f_{7}=-\sum_{k=2}^{2Ng} p_{i+j,k}{log}_{2}p_{i+j,k}$$

Entropy_GLCM_.( Joint entropy) is defined as:

$$f_{8}=-\sum_{i=1}^{Ng} \sum_{j=1}^{Ng} p_{i,j}{log}_{2}p_{i,j}$$

Difference Variance (DVAR): The difference variance for the diagonal probabilities

$$f_{9}=-\sum_{k=0}^{Ng-1} \left( k-\mu\right)^{2}{\cdot p}_{i-j,k}$$

GLCM Difference Entropy (DENT): The difference entropy for the diagonal probabilities

$$f_{10}=-\sum_{k=0}^{Ng-1} p_{i-j,k}{log}_{2}p_{i-j,k}$$

Information Correlation (IC): set to infinity if the denominator is zero.

$$f_{11}=-\sum_{i=1}^{Ng} \sum_{j=1}^{Ng} {p_{i.}p_{.j}log}_{2}\left( p_{i.}p_{.j} \right)$$

Autocorrelation is defined as:

$$f_{12}=\sum_{i=1}^{Ng} \sum_{j=1}^{Ng} i\cdot j\cdot p_{i,j}$$

Dissimilarity is conceptually similar to the contrast feature

$$f_{13}=\sum_{i=1}^{Ng} \sum_{j=1}^{Ng} \left| i-j \right|\cdot p_{i,j}$$

Cluster Prominence (CP) is defined as:

$$f_{14}=\sum_{i=1}^{Ng} \left. \sum_{j=1}^{Ng} \left. \left( i+j-\mu_{i}-\mu_{j} \right)^{4}\cdot p_{i,j} \right. \right.$$

Joint maximum is defined as:

$$f_{15}=\max\left\{ p_{i,j} \right\}$$

Inverse Difference (ID) is a measure of homogeneity. Grey level co-occurrences with a large difference in levels are weighed less, thus lowering the total feature value. The feature score is maximal if all grey levels are the same.

$$f_{16}=\sum_{i=1}^{Ng} \left. \sum_{j=1}^{Ng} \frac{1}{1+\left| i-j \right|}\cdot p_{i,j} \right.$$

1. **Grey Level Size Zone Matrix (GLSZM)**

Let *p* be the grey level size zone matrix (GLSZM) indexed by *p_i,j_* with rows *i* indicating grey levels and columns *j* indicating zone sizes. The largest zone size (the number of columns) will be denoted *S_max_*. The total number of unique connected zones is *n_z_*. The total number of voxels is *n_v_*.

Small Zone Emphasis (SZSE): This feature emphasizes small zones.

$$Z_{1}=\frac{1}{Ns}\sum_{j=1}^{Nz} \frac{s_{j}}{j^{2}}$$

Large Zone Emphasis (LZSE): This feature emphasizes large zones.

$$Z_{2}=\frac{1}{Ns}\sum_{j=1}^{Nz} j^{2}s_{j}$$

Low Grey Level Zone Emphasis (LGLZE): This feature is a grey level analogue to small zone emphasis.

$$Z_{3}=\frac{1}{Ns}\sum_{i=1}^{Ng} \frac{s_{i}}{i^{2}}$$

High Grey Level Zone Emphasis (HGLZE): The high grey level zone emphasis feature is a grey level analogue to large zone emphasis.

$$Z_{4}=\frac{1}{Ns}\sum_{i=1}^{Ng} i^{2}s_{i}$$

Small Zone Low Grey Level Emphasis (SZLGE): This feature emphasizes zone counts within the upper left quadrant of the GLSZM, where small zone sizes and low grey levels are located.

$$Z_{5}=\frac{1}{N_{s}}\cdot\sum_{i=1}^{N_{g}} \sum_{j=1}^{N_{Z}} \left. \frac{s_{i,j}}{i^{2}\cdot j^{2}} \right.$$

Small Zone High Grey Level Emphasis (SZHGE): This feature emphasizes zone counts in the lower left quadrant of the GLSZM, where small zone sizes and high grey levels are located.

$$Z_{6}=\frac{1}{N_{s}}\cdot\sum_{i=1}^{N_{g}} \sum_{j=1}^{N_{z}} \frac{s_{i,j}\cdot i^{2}}{j^{2}}$$

Large Zone Low Grey Level Emphasis (LZLGE): This feature emphasizes zone counts in the upper right quadrant of the GLSZM, where large zone sizes and low grey levels are located.

$$Z_{7}=\frac{1}{N_{s}}\cdot\sum_{i=1}^{N_{g}} \sum_{j=1}^{N_{z}} \frac{s_{i,j}\cdot j^{2}}{i^{2}}$$

Large Zone High Grey Emphasis (LZHGE): This feature emphasizes zone counts in the lower right quadrant of the GLSZM, where large zone sizes and high grey levels are located.

$$Z_{8}=\frac{1}{N_{s}}\cdot\sum_{i=1}^{N_{g}} \sum_{j=1}^{N_{z}} s_{i,j}\cdot i^{2}\cdot j^{2}$$

Gray-Level Non-Uniformity (GLNU) represents the similarity of intensity values in an image

$$Z_{9}=\frac{1}{Ns}\sum_{i=1}^{Ng} {s_{i}}^{2}$$

Zone Size Non-Uniformity (ZSNU): This feature assesses the distribution of zone counts over the different zone sizes. Zone size non-uniformity is low when zone counts are equally distributed along zone sizes.

$$Z_{10}=\frac{1}{Ns}\sum_{i=1}^{Ng} {s_{j}}^{2}$$

Zone Size Percentage (SZP): This feature assesses the fraction of the number of realized zones and the maximum number of potential zones. Highly uniform ROIs produce a low zone percentage.

$$Z_{11}=\frac{N_{s}}{N_{v}}$$

References:

1. Parmar C, Velazquez ER, Leijenaar R, Jermoumi M, Carvalho S, Mak RH, et al. Robust radiomics feature quantification using semiautomatic volumetric segmentation. PLoS One. 2014;9(7):1–8.

2. Zwanenburg, Alex; Leger, Stefan; Vallières, Martin; Löck, Steffen; Image Biomarker Standardisation Initiative for the. Image biomarker standardisation initiative. eprint arXiv:161207003 [Internet]. Available from: 2016arXiv161207003Z

3. Aerts HJ, Velazquez ER, Leijenaar RT, Parmar C, Grossmann P, Carvalho S, et al. Decoding tumour phenotype by noninvasive imaging using a quantitative radiomics approach. Nat Commun. 2014;5:4006.

4. Ganeshan B, Skogen K, Pressney I, Coutroubis D, Miles K. Tumour heterogeneity in oesophageal cancer assessed by CT texture analysis: Preliminary evidence of an association with tumour metabolism, stage, and survival. Clin Radiol. 2012;67(2):157–64.

5. Bogdan Badic, Marie Charlotte Desseroit, Mathieu Hatt DV. Potential Complementary Value of Noncontrast and Contrast Enhanced. Acad Radiol [Internet]. 2018;1–11. Available from: https://doi.org/10.1016/j.acra.2018.06.004

6. Hatt M, Tixier F, Pierce L, Kinahan PE, Le Rest CC VD. Characterization of PET/CT images using texture analysis: the past, the present… any future? Eur J Nucl Med Mol Imaging.

7. Hatt M, Tixier F, Pierce L, Kinahan PE, Le Rest CC, Visvikis D. Characterization of PET/CT images using texture analysis: the past, the present… any future? Eur J Nucl Med Mol Imaging. 2017;44(1):151–65.

8. Alex Zwanenburg, Stefan Leger, Martin Vallières SL. Image biomarker standardisation initiative. Reference manual. 2016; Available from: https://www.cancerdata.org/resource/doi:10.17195/candat.2016.08.1

9. No Title [Internet]. Available from: https://portal.gdc.cancer.gov

10. No Title [Internet]. Available from: https://gdac.broadinstitute.org

11. Network TCGA, Muzny DM, Bainbridge MN, Chang K, Dinh HH, Drummond JA, et al. Comprehensive molecular characterization of human colon and rectal cancer. Nature [Internet]. 2012 Jul 18;487:330. Available from: https://doi.org/10.1038/nature11252

## Supplementary material 2

To establish an independent validation set, we have downloaded expression microarray and RNA-sequencing data from 221 and 358 CRC patients, respectively, from The Cancer Genome Atlas (TCGA) portal(9) and the Broad GDAC Firehose(10) by selecting the colorectal adenocarcinoma cohort (COADREAD). The mRNA expression levels (TCGA Level III) of candidate genes were investigating using two different sets of samples according two transcriptomic platforms: i) 221 CRC and 22 normal colon tissues by Custom Agilent 244K Gene Expression Microarray and log2 transformed-lowess normalized values, ii) 358 CRC and 51 normal colon tissues by Illumina HiSeq2000 RNA-sequencing platform and normalized counts by RSEM algorithm(11). Noted that two sets of expression data are different with only 4 tumors and 21 normal tissues processed by the 2 methods.

Characteristics of patients included in TCGA analysis were displayed in Supplementary Table 1. Repartition of low-stage (I and II) and high-stage (III and IV) was homogeneous in RNA-seq set of TCGA, while microarray set contains more low-stage (60% low versus 40% high-stage).

**References:**

1. Parmar C, Velazquez ER, Leijenaar R, Jermoumi M, Carvalho S, Mak RH, et al. Robust radiomics feature quantification using semiautomatic volumetric segmentation. PLoS One. 2014;9(7):1–8.

2. Zwanenburg, Alex; Leger, Stefan; Vallières, Martin; Löck, Steffen; Image Biomarker Standardisation Initiative for the. Image biomarker standardisation initiative. eprint arXiv:161207003 [Internet]. Available from: 2016arXiv161207003Z

3. Aerts HJ, Velazquez ER, Leijenaar RT, Parmar C, Grossmann P, Carvalho S, et al. Decoding tumour phenotype by noninvasive imaging using a quantitative radiomics approach. Nat Commun. 2014;5:4006.

4. Ganeshan B, Skogen K, Pressney I, Coutroubis D, Miles K. Tumour heterogeneity in oesophageal cancer assessed by CT texture analysis: Preliminary evidence of an association with tumour metabolism, stage, and survival. Clin Radiol. 2012;67(2):157–64.

5. Bogdan Badic, Marie Charlotte Desseroit, Mathieu Hatt DV. Potential Complementary Value of Noncontrast and Contrast Enhanced. Acad Radiol [Internet]. 2018;1–11. Available from: https://doi.org/10.1016/j.acra.2018.06.004

6. Hatt M, Tixier F, Pierce L, Kinahan PE, Le Rest CC VD. Characterization of PET/CT images using texture analysis: the past, the present… any future? Eur J Nucl Med Mol Imaging.

7. Hatt M, Tixier F, Pierce L, Kinahan PE, Le Rest CC, Visvikis D. Characterization of PET/CT images using texture analysis: the past, the present… any future? Eur J Nucl Med Mol Imaging. 2017;44(1):151–65.

8. Alex Zwanenburg, Stefan Leger, Martin Vallières SL. Image biomarker standardisation initiative. Reference manual. 2016; Available from: https://www.cancerdata.org/resource/doi:10.17195/candat.2016.08.1

9. No Title [Internet]. Available from: https://portal.gdc.cancer.gov

10. No Title [Internet]. Available from: https://gdac.broadinstitute.org

11. Network TCGA, Muzny DM, Bainbridge MN, Chang K, Dinh HH, Drummond JA, et al. Comprehensive molecular characterization of human colon and rectal cancer. Nature [Internet]. 2012 Jul 18;487:330. Available from: https://doi.org/10.1038/nature11252

**Supplementary Table 1.** Clinical and histological characteristics of COADREAD TCGA cohort.

Transcriptome analysis was been evaluated in two different sets of samples, according microarray and RNA-seq technologies.

|  | **Microarray gene expression profiling** | **RNAseq gene expresion profiling** |
| --- | --- | --- |
| **Normal Colon Tissue** | n=22 | n=51 |
| **Colorectal Carcinoma** | n= 221 | n= 358 |
| **Gender (male/female)** | 115 / 106 | 197 / 161 |
| **Tumor tissue site** | 151 colon; 68 rectum; 2 NA | 274 colon ; 82 rectum ; 2 NA |
| **Mean age (years) at initial diagnosis** | 69.5 (range: 35-90) | 64.5 (range: 31-90) |
| **Dead/Alive** | 181 / 40 | 79 / 279 |
| **Stage I** | 47 | 57 |
| **Stage II** | 85 | 136 |
| **Stage III** | 55 | 113 |
| **Stage IV** | 34 | 52 |

NA-not available.

**Supplementary Table 3 *Characteristics and functions of the gene panel***

| **Used NAME** | **Accession Number** | **Sequence Definition** | **Sequence Length** | **Product Length** | **Sense Primer** | **Anti-sense Primer** |
| --- | --- | --- | --- | --- | --- | --- |
|  |  |  |  |  |  |  |
| **ABCB1** | NM_000927 | Homo sapiens ATP binding cassette subfamily B member 1 (ABCB1), mRNA. | 4.718 | 129 | CTGAGCCTGGAGGTGAAGAAG | GCCATCAAGCAGCACTTTCC |
| **ABCC2** | NM_000392 | Homo sapiens ATP binding cassette subfamily C member 2 | 5.446 | 107 | GCCTACTCCTGCCTGTTCT | GCTATGGATGATGGATTATTTGATGAC |
| **ABCG2** | NM_001257386 | Homo sapiens ATP binding cassette subfamily G member 2 | 4.479 | 129 | GCCTCTATAGCTCAGATCATTGTCA | GGTTGGTCGTCAGGAAGAAGA |
| **ALCAM** | NM_001627 | Homo sapiens activated leukocyte cell adhesion molecule | 4.923 | 125 | CTACAATAGTCAAGGTGTTCAAGCAA | GCCATCTGGATAACTGTCTTCTGA |
| **ALDH1A1** | NM_000689 | Homo sapiens aldehyde dehydrogenase 1 family member A1 | 2.378 | 97 | CATGATTCAGTGAGTGGCAAGA | TGTCAACATCCTCCTTATCTCCTT |
| **B2M** | NM_004048 | Homo sapiens beta-2-microglobulin (B2M), mRNA. | 987 | 133 | GGCTATCCAGCGTACTCCAA | TCCATTCTTCAGTAAGTCAACTTCAAT |
| **CDKN1Av1** | NM_000389 | Homo sapiens cyclin dependent kinase inhibitor 1A (CDKN1A), transcript variant 1, mRNA. | 2.175 | 112 | CCCTTGTGCCTCGCTCAG | CGTTTGGAGTGGTAGAAATCTGTCAT |
| **CDKN1Av2** | NM_078467 | Homo sapiens cyclin dependent kinase inhibitor 1A (CDKN1A), transcript variant 2, mRNA. | 2.122 | 143 | ACCAGCATGACAGATTTCTACCA | AACTGAGACTAAGGCAGAAGATGT |
| **INHBB** | NM_002193 | Homo sapiens inhibin beta B subunit (INHBB), mRNA. | 3.218 | 124 | TTCGCCGAGACAGATGGC | AGGGCAGGAGTTTCAGGTAAAG |
